# Burden of multimorbidity, socioeconomic status and use of health services across stages of life in urban areas: a cross-sectional study

**DOI:** 10.1186/1471-2458-14-530

**Published:** 2014-05-29

**Authors:** Concepción Violán, Quintí Foguet-Boreu, Albert Roso-Llorach, Teresa Rodriguez-Blanco, Mariona Pons-Vigués, Enriqueta Pujol-Ribera, Miguel Ángel Muñoz-Pérez, Jose M Valderas

**Affiliations:** 1 Institut Universitari d’Investigació en Atenció Primària Jordi Gol (IDIAP Jordi Gol), Barcelona, Spain; 2 Universitat Autònoma de Barcelona, Bellaterra (Cerdanyola del Vallès), Barcelona, Spain; 3 Institut Català de la Salut, Barcelona, Catalunya, Spain; 4 Hospital de Campdevànol, Campdevànol, Spain; 5 Health Services & Policy Research Group, School of Medicine, University of Exeter, Exeter EX1 2 LU, UK

**Keywords:** Multimorbidity, Chronic conditions, Socioeconomic status, Use of health services, Life-stage, Urban area, Inequalities

## Abstract

**Background:**

The burden of chronic conditions and multimorbidity is a growing health problem in developed countries. The study aimed to determine the estimated prevalence and patterns of multimorbidity in urban areas of Catalonia, stratified by sex and adult age groups, and to assess whether socioeconomic status and use of primary health care services were associated with multimorbidity.

**Methods:**

A cross-sectional study was conducted in Catalonia. Participants were adults (19+ years) living in urban areas, assigned to 251 primary care teams. Main outcome: multimorbidity (≥2 chronic conditions). Other variables: sex (male/female), age (19–24; 25–44; 45–64; 65–79; 80+ years), socioeconomic status (quintiles), number of health care visits during the study.

**Results:**

We included 1,356,761 patients; mean age, 47.4 years (SD: 17.8), 51.0% women. Multimorbidity was present in 47.6% (95% CI 47.5-47.7) of the sample, increasing with age in both sexes but significantly higher in women (53.3%) than in men (41.7%). Prevalence of multimorbidity in each quintile of the deprivation index was higher in women than in men (except oldest group). In women, multimorbidity prevalence increased with quintile of the deprivation index. Overall, the median (interquartile range) number of primary care visits was 8 (4–14) in multimorbidity vs 1 (0–4) in non-multimorbidity patients. The most prevalent multimorbidity pattern beyond 45 years of age was uncomplicated hypertension and lipid disorder. Compared with the least deprived group, women in other quintiles of the deprivation index were more likely to have multimorbidity than men until 65 years of age. The odds of multimorbidity increased with number of visits in all strata.

**Conclusions:**

When all chronic conditions were included in the analysis, almost 50% of the adult urban population had multimorbidity. The prevalence of multimorbidity differed by sex, age group and socioeconomic status. Multimorbidity patterns varied by life-stage and sex; however, circulatory-endocrine-metabolic patterns were the most prevalent multimorbidity pattern after 45 years of age. Women younger than 80 years had greater prevalence of multimorbidity than men, and women’s multimorbidity prevalence increased as socioeconomic status declined in all age groups. Identifying multimorbidity patterns associated with specific age-related life-stages allows health systems to prioritize and to adapt clinical management efforts by age group.

## Background

Demographers predict a 70% increase in the aging population by 2050 in the more developed regions of the world [1]. Accompanied by a predictable increase in multimorbidity, defined as the coexistence of two or more chronic conditions in the same person at one point in time, this shift will present a challenge for health systems. Knowledge of the prevalence and patterns of multimorbidity, stratified by age and sex, will be needed to deliver high-quality health care adapted to patient needs.

To date, few studies have analysed the distribution of multimorbidity across the different stages of life [2-7]. On the other hand, the number of primary care visits is clearly associated with a patient’s number of chronic conditions [8,9] and has been used as a proxy for the burden of multimorbidity in this setting. Furthermore, there is evidence linking socioeconomic status and multimorbidity [2,10,11]. However, no studies have evaluated all these aspects in urban populations. The concentration of most of the world’s population in urban areas, the establishment of the urban setting as a determinant of health, and the inclusion of this concern among the research priorities of international health organizations [12-14] led to the design of this study of multimorbidity with a focus on the urban environment.

The study aimed to determine the estimated prevalence and patterns of multimorbidity in urban areas of Catalonia, stratified by sex and adult age groups, and to assess whether socioeconomic status and use of primary health care services were associated with multimorbidity.

## Methods

### Design, setting and study population

**Figure 1:**
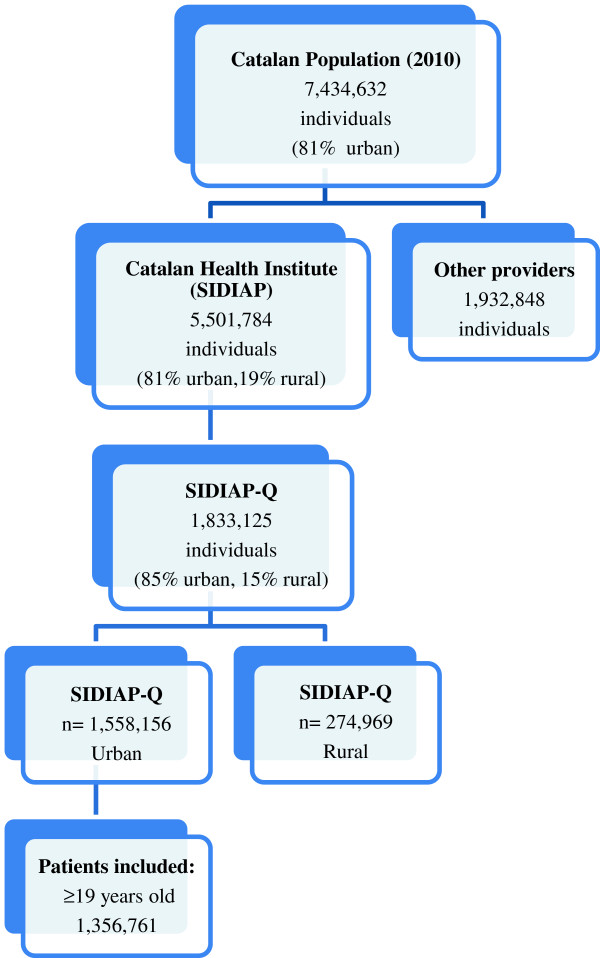
Flow chart of patients included in the study.

A cross-sectional study was conducted in Catalonia (Spain), a Mediterranean region (southern Europe) with 7,434,632 inhabitants, 81% of which live in urban municipalities (2010 census). The Spanish National Health Service (NHS) provides universal coverage, financed mainly by tax revenue. The Catalan Health Institute (CHI) manages primary health care teams (PHCT) that serve 5,501,784 patients; the remaining PHCT are managed by other providers. The CHI’s Information System for the Development of Research in Primary Care (SIDIAP) contains the coded clinical information recorded in electronic health records (EHR) by its 274 PHCT [15]. A subset of records meeting the highest quality criteria for clinical data (SIDIAP Q) includes 40% of the SIDIAP population (1,833,125 patients), attended by the 1,365 general practitioners (GPs) whose data recording scored highest in a validated comparison process [16]. Of the 85% of all SIDIAP Q patients who live in urban areas, we selected patients aged 19 years or older assigned to 251 PHCT on 31 December 2010 (Figure 1). The criteria for defining an urban area were 10,000 or more inhabitants and a population density of at least 150 residents/km^2^[15].

### Clinical information and variables

International Classification of Diseases (ICD-10) codes were mapped to International Classification of Primary Care (ICPC-2) codes. Each diagnosis was then classified using O’Halloran criteria for chronic conditions. We included all 146 diagnoses considered as chronic conditions by O’Halloran criteria: (i) have a duration that has lasted, or is expected to last, at least 6 months; (ii) have a pattern of recurrence or deterioration; (iii) have a poor prognosis and (iv) produce consequences, or sequelae, that have an impact on the individual’s quality of life [17,18].

Multimorbidity was defined as the coexistence of 2 or more chronic conditions recorded in the EHR as of 31 December 2010. All results were described with ICPC-2 codes.

The main outcome was the presence of multimorbidity (yes/no). Other variables were sex (male/female); age (young adult, 19 to 24; adult, 25–44; older adult, 45–64; elderly, 65–79; oldest adult, 80+); socioeconomic status (deprivation quintiles: Q1, least deprived, to Q5, most deprived); number of visits during previous 12 months, categorized as continuous or as no visits; and quartiles of attended population (0, 1–2, 3–5, 6–10, ≥11).

In urban areas, deprivation indexes have been shown to be useful as a proxy variable for socioeconomic status [19]. Dominguez-Berjon et al. describe the deprivation index defined in the MEDEA Project and selected for use in the present study. This deprivation index calculates socioeconomic status in urban areas for each patient’s area of residence according to 5 indicators available for each census tract (unit of area of residence) in 2001: unemployment (≥16 years old), low educational level overall (≥16 years old), low educational level in young adults (16–29 years old), workers with manual jobs, and temporary workers. Higher scores on this index represent greater socioeconomic deprivation [20]. The construction of the index applied the factor loadings of the principal components analysis of Barcelona city to the other Catalan urban areas for two reasons. First, several socioeconomic indicators of the index were unavailable for each census tract in these other urban areas. Second, we determined that, adjusted for age and sex, the main diseases recorded in primary care, by quintiles of the deprivation index in the urban areas throughout Catalonia, followed a pattern similar to that of Barcelona city.

Number of visits was used as a proxy of use of health services. This included patient visits to the primary care centre and home health care visits recorded in the EHR by a GP, a nurse or social worker.

### Statistical analysis

Analysis was stratified by sex and age group. Descriptive statistics were used to summarize overall information. Categorical variables were expressed as frequencies (percentage) and continuous as the median (interquartile range, IQR). The prevalence of multimorbidity (2 or more chronic conditions) was calculated for each stratum.

The differences between groups were tested using Student t, Mann–Whitney, Chi square or Fisher exact test for unadjusted comparison, as appropriate. Correlation between variables was measured by the Pearson or Spearman correlation coefficient.

Prevalence estimates of each chronic condition and 95% confidence intervals (CI) were obtained. The 10 chronic conditions with the highest prevalence, stratified by sex, and the number of chronic conditions associated with each disease were computed.

To determine the most prevalent multimorbidity patterns, all possible combinations of any 2 and 3 chronic conditions and their frequencies were calculated. To synthesize the results, we present only the most frequent combinations.

A multilevel logistic regression model was used to identify the factors associated with multimorbidity and study the association between the presence of each chronic condition and multimorbidity/non-multimorbidity. The PHCT was a random variable. The abovementioned co-variables were considered as independent variables.

All statistical tests were two-sided at the 5% significance level. The analyses were performed using SPSS for Windows, version 18 (SPSS Inc., Chicago, IL, USA), Stata/SE version 11 for Windows (Stata Corp. LP, College Station, TX, USA) and R version 2.15.2 (R Foundation for Statistical Computing, Vienna, Austria).

### Ethical considerations

The study protocol was approved by the Committee on the Ethics of Clinical Research, Institut Universitari d’Investigació en Atenció Primària (IDIAP) Jordi Gol (Protocol No: P12/28). All data were anonymized and the confidentiality of EHR was respected at all times in accordance with international law.

## Results

We included 1,356,761 patients. Mean age was 47.4 years (standard deviation [SD]: 17.8) and 51.0% were women. Multimorbidity was present in 47.6% (95% CI, 47.5-47.7) of the sample, increasing with age in both sexes but significantly higher in women (53.3%) than in men (41.7%) (p < 0.001).

Greater multimorbidity prevalence was observed in women than in men in each quintile of the deprivation index, except in the oldest age group (80+ years). In women, multimorbidity prevalence increased as women’s socioeconomic status declined in all age groups. Under the age of 65 years, there was greater variation in multimorbidity prevalence among women than men across all quintiles of the deprivation index (Table 1).

**Table 1:** Estimated prevalence of multimorbidity within quintiles of deprivation index and strata of number of visits in 2010, stratified by sex and age group (N = 1,356,761)

	**Women**				
	**19-24 years**	**25-44 years**	**45-64 years**	**65-79 years**	**80+ years**
	**n = 52,222 (MM: 19.6%)**	**n = 276,275 (MM: 30.0%)**	**n = 209,969 (MM: 64.4%)**	**n = 105,682 (MM: 91.2%)**	**n = 47,498 (MM: 93.3%)**
Deprivation index					
Least deprived	15.7	25.0	56.5	87.7	90.9
Q2	18.5	28.4	62.0	90.3	93.2
Q3	20.6	30.8	66.0	91.8	93.2
Q4	20.9	31.8	68.7	92.8	94.6
Most deprived	21.8	34.1	70.6	93.3	95.2
Number of visits					
*Median (IQR)*	6 (3–10)	6 (3–11)	8 (4–13)	11 (7–18)	14 (8–23)
0	6.8	10.5	19.9	33.5	45.1
1-2	13.0	21.3	46.4	70.2	75.8
3-5	20.3	31.0	64.9	86.8	89.7
6-10	29.1	44.0	79.3	94.5	95.3
≥11	41.8	61.3	90.8	98.4	98.0
	**Men**				
	**19-24 years**	**25-44 years**	**45-64 years**	**65-79 years**	**80+ years**
	**n = 54,094 (MM: 13.3%)**	**n = 297,127 (MM: 19.9%)**	**n = 200,261 (MM: 54.7%)**	**n = 87,504 (MM: 88.0%)**	**n = 26,129 (MM: 93.0%)**
Deprivation index					
Least deprived	11.6	17.4	49.3	84.4	91.0
Q2	11.8	19.3	53.9	87.5	92.6
Q3	14.4	20.3	55.8	88.3	93.4
Q4	14.2	20.8	57.7	89.6	94.2
Most deprived	14.0	21.1	57.6	90.0	93.8
Number of visits					
*Median (IQR)*	*4 (1–8)*	*5 (2–9)*	*6 (3–11)*	*11 (6–17)*	*14 (8–23)*
0	5.7	7.2	18.0	31.9	38.3
1-2	11.0	15.9	42.7	65.4	73.1
3-5	16.3	25.1	61.2	83.5	88.0
6-10	24.0	36.5	76.1	92.7	95.1
≥11	33.8	54.2	88.3	97.4	97.7

*Abbreviations*: *n* total number of patients in each stratum, *MM* multimorbidity, *Qi* quintiles of deprivation (Q1, least deprived to Q5, most deprived); *IQR* interquartile range.

Data are expressed as percentage, unless otherwise stated.

Overall, the median (interquartile ratio [IQR]) number of visits was 8[4-14] in patients with multimorbidity vs. 1[0–4] in the non-multimorbidity group. Among patients with multimorbidity, the median number of visits was higher among women than men until 65 years of age, but became more equalized in the older age groups (Table 1).

The number of chronic conditions increased with age, and was higher in women than in men (Table 2). In both sexes, lipid disorder and uncomplicated hypertension were the most common of the top 10 and systematically coexisted with some other chronic condition (Table 3).

**Table 2:** Distribution of number of chronic conditions in 2010, by sex and age group (N = 1,356,761)

	**Women**				
	**19-24 years**	**25-44 years**	**45-64 years**	**65-79 years**	**80+ years**
	**n = 52,222**	**n = 276,275**	**n = 209,969**	**n = 105,682**	**n = 47,498**
Number of conditions					
0	52.3	42.9	18.4	4.0	3.5
1	28.1	27.1	17.2	4.7	3.2
2	12.4	15.4	16.7	8.2	5.9
3	4.8	7.8	14.4	11.6	8.6
4	1.6	3.7	11.1	13.1	11.4
≥5	0.8	3.1	22.2	58.4	67.4
	**Men**				
	**19-24 years**	**25-44 years**	**45-64 years**	**65-79 years**	**80+ years**
	**n = 54,094**	**n = 297,127**	**n = 200,261**	**n = 87,504**	**n = 26,129**
Number of conditions					
0	61.4	55.4	25.9	5.3	3.7
1	25.4	24.7	19.4	6.6	3.3
2	9.1	11.3	16.8	10.4	6.0
3	2.9	5.0	13.2	13.3	9.3
4	0.8	2.1	9.5	14.4	11.7
≥5	0.4	1.5	15.2	50.0	66.0

*Abbreviation*: *n* total number of patients in each stratum.

Data are expressed as percentage of total patients within each category considered.

**Table 3:** Prevalence of top 10 chronic conditions and number of associated chronic conditions in 2010, stratified by sex (N = 1,356,761)

**Women (n = 691,646)**							
				Comorbidity*			
**ICPC2**	**Chronic conditions**	**n†**	**Alone**	**+1**	**+2**	**+3**	**+4 or more**
T93	Lipid disorder	155,451	5.7	10.8	13.6	14.0	55.9
K86	Hypertension, uncomplicated	150,293	3.2	7.8	11.5	13.6	63.9
P76	Depressive disorder	99,979	9.7	13.4	13.9	12.8	50.2
T82	Obesity	90,376	7.7	11.4	12.7	12.8	55.4
P74	Anxiety disorder/anxiety state	83,866	16.8	19.5	16.3	12.3	35.1
K95	Varicose veins of leg	64,319	7.5	10.8	11.8	12.0	57.9
L91	Osteoarthrosis, other	55,643	2.3	5.2	8.5	11.3	72.7
L95	Osteoporosis	51,807	2.8	6.5	10.2	12.4	68.1
L86	Back syndrome with radiating pain	48,594	10.2	12.8	12.9	11.9	52.2
T90	Diabetes, non-insulin dependent	47,816	1.5	4.2	7.8	11.3	75.2
**Men (n = 665,115)**							
				Comorbidity*			
**ICPC2**	**Chronic conditions**	**n†**	**Alone**	**+1**	**+2**	**+3**	**+4 or more**
T93	Lipid disorder	134,292	10.9	15.8	16.5	14.7	42.1
K86	Hypertension, uncomplicated	125,826	6.1	11.8	15.0	15.5	51.6
T90	Diabetes, non-insulin dependent	53,264	3.3	8.0	12.3	14.7	61.7
T82	Obesity	52,682	10.9	14.7	15.1	14.0	45.3
Y85	Benign prostatic hypertrophy	47,032	3.2	7.4	11.4	13.7	64.3
P74	Anxiety disorder/anxiety state	40,619	23.0	22.3	16.8	11.8	26.1
L86	Back syndrome with radiating pain	35,487	17.6	16.7	14.4	12.0	39.3
P76	Depressive disorder	34,709	14.1	16.8	15.3	12.8	41.0
T99	Endocr/metab/nutrit disease, other	28,256	5.8	10.1	12.9	13.4	57.8
P15	Chronic alcohol abuse	26,527	14.3	17.8	17.1	14.1	36.7

*Abbreviations*: *ICPC 2* International Classification of Primary Care, *n†* total number of patients with each chronic condition, *Endocr/metab/nutrit disease* Endocrine, metabolic, nutritional disease. Data are expressed as percentage.

*Numbers indicate the percentage of each chronic condition considered which coexisted with another chronic condition (+1), 2 more chronic conditions (+2), 3 more chronic conditions (+3) or with 4 or more chronic conditions (+4 or more).

In the analysis by age and sex, the prevalence of the most common chronic conditions differed by sex in younger adults: anxiety disorder/anxiety state (women aged 19–44), acne (men aged 19–24) and lipid disorder (men aged 25–44). Among older adults, the most prevalent chronic conditions did not differ by sex: lipid disorder (aged 45–64) and uncomplicated hypertension (aged 65 and older) (Additional file 1).

The most prevalent pairs of chronic conditions in women consistently included anxiety disorder/anxiety state in participants aged 19 to 44 years. The most prevalent pairs of chronic conditions did not differ by sex after 45 years of age, when uncomplicated hypertension and lipid disorder became the most common combination (Table 4).

**Table 4:** Most prevalent multimorbidity patterns in 2010, by age group and sex (N = 1,356,761)

			**Pairs**		**Triplets**	
**Sex**	**Age groups (years)**	**n**	**Chronic conditions**	**%**	**Chronic conditions**	**%**
**Women**	19-24	52,222	Anxiety disorder/anxiety state & Acne	0.9	Anxiety disorder/anxiety state & Acne & Depressive disorder	0.1
	25-44	276,275	Anxiety disorder/anxiety state & Depressive disorder	1.9	Anxiety disorder/anxiety state & Depressive disorder & Obesity	0.2
	45-64	209,969	Hypertension, uncomplicated & Lipid disorder	10.1	Hypertension, uncomplicated & Lipid disorder & Obesity	3.5
	65-79	105,682	Hypertension, uncomplicated & Lipid disorder	36.0	Hypertension, uncomplicated & Lipid disorder & Obesity	11.6
	80+	47,498	Hypertension, uncomplicated & Lipid disorder	37.7	Hypertension, uncomplicated & Lipid disorder & Diabetes (non-insulin dependent)	11.2
**Men**	19-24	54,094	Acne & Asthma	0.7	Acquired deformity of spine & Asthma & Acne	0.1
	25-44	297,127	Lipid disorder & Obesity	1.1	Hypertension, uncomplicated & Lipid disorder & Obesity	0.3
	45-64	200,261	Hypertension, uncomplicated & Lipid disorder	12.3	Hypertension, uncomplicated & Lipid disorder & Diabetes (non-insulin dependent)	3.4
	65-79	87,504	Hypertension, uncomplicated & Lipid disorder	29.7	Hypertension, uncomplicated & Lipid disorder & Diabetes (non-insulin dependent)	10.8
	80+	26,129	Hypertension, uncomplicated & Lipid disorder	26.9	Hypertension, uncomplicated & Lipid disorder & Benign prostatic hypertrophy	10.9

Data are expressed as percentage.

*Abbreviation*: *n* total number of patients in each stratum.

**Figure 2:**
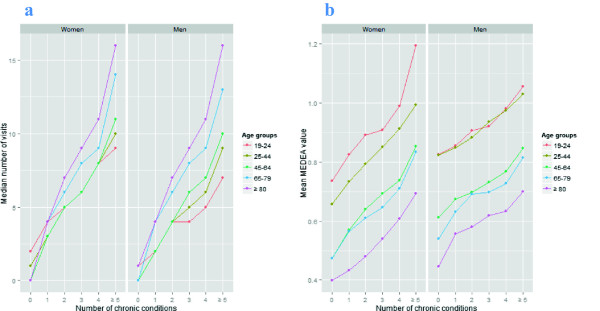
**Relation between number of chronic conditions and median number of visits (2a) and the mean deprivation value (2b), stratified by age group (years) and sex.** In this figure, the X-axis shows the number of chronic conditions in both figures. The Y-axis shows the median number of visits** (2a)** and mean value on the deprivation index (0.4, Least deprived; 1.2, most deprived) **(2b)** by sex and age group (colour-coded lines).

The highest odds ratios (ORs) of multimorbidity vs. non-multimorbidity were observed as follows: acquired deformity of spine (women and men ages 19–24), obesity (women and men ages 25 to 79), osteoarthrosis (women aged 80+) and lipid disorder (men aged 80+) (Table 5).The relationship between number of chronic conditions and median number of health care visits and the mean deprivation value by age group and sex is shown in Figure 2. In both sexes, visits and socioeconomic de privation increased as the number of chronic conditions increased. The entire sample showed a positive correlation between the number of chronic conditions and the number of visits (rho Spearman = 0.63, P < 0.001).

**Table 5:** The highest odds ratio of chronic conditions of multimorbidity vs. non-multimorbidity, by sex and age group

**Age groups (years)**	**Women**						**Men**					
	**ICPC 2**	**Chronic conditions**	**Prevalence**		**OR**	**95% CI**	**ICPC 2**	**Chronic conditions**	**Prevalence**		**OR**	**95% CI**
			**MM**	**Non-MM**					**MM**	**Non-MM**		
19-24	L85	Acquired deformity of spine	15.1%	2.3%	7.7	(7.0-8.4)	L85	Acquired deformity of spine	14.3%	1.5%	11.2	(10.0-12.4)
	T82	Obesity	16.0%	2.4%	7.4	(6.8-8.1)	T82	Obesity	15.2%	1.8%	9.7	(8.8-10.8)
	P74	Anxiety disorder/anxiety state	27.7%	4.0%	6.9	(6.5-7.4)	P74	Anxiety disorder/anxiety state	16.5%	1.9%	7.7	(7.0-8.5)
	S96	Acne	23.0%	4.4%	6.2	(5.8-6.7)	R96	Asthma	23.4%	3.6%	7.4	(6.9-8.0)
	R96	Asthma	17.0%	2.8%	6.2	(5.7-6.8)	S96	Acne	28.7%	4.9%	7.3	(6.8-7.9)
25-44	T82	Obesity	18.0%	2.2%	8.9	(8.6-9.2)	T82	Obesity	16.3%	1.5%	12.5	(12.0-13.1)
	T93	Lipid disorder	13.3%	1.6%	8.4	(8.1-8.8)	T93	Lipid disorder	26.5%	2.8%	10.7	(10.4-11.1)
	P76	Depressive disorder	22.5%	3.0%	7.9	(7.6-8.1)	P76	Depressive disorder	13.2%	1.3%	9.1	(8.7-9.5)
	N89	Migraine	12.2%	1.8%	6.6	(6.3-6.9)	P74	Anxiety disorder/anxiety state	21.8%	2.9%	7.0	(6.8-7.2)
	P74	Anxiety disorder/anxiety state	29.8%	5.0%	6.3	(6.1-6.5)	L86	Back syndrome with radiating pain	10.8%	1.7%	5.6	(5.3-5.8)
45-64	T82	Obesity	23.2%	2.2%	11.0	(10.5-11.7)	T82	Obesity	18.4%	1.5%	13.7	(12.9-14.6)
	K86	Hypertension, uncomplicated	31.6%	3.3%	8.8	(8.4-9.2)	K86	Hypertension uncomplicated	41.5%	4.7%	9.6	(9.3-10.0)
	T93	Lipid disorder	42.2%	6.0%	8.5	(8.2-8.8)	T90	Diabetes non-insulin dependent	18.2%	1.2%	9.5	(8.9-10.1)
	P76	Depressive disorder	27.5%	4.2%	6.3	(6.1-6.6)	T93	Lipid disorder	49.8%	7.7%	9.4	(9.2-9.7)
	P74	Anxiety disorder/anxiety state	19.5%	3.5%	5.2	(4.9-5.4)	L86	Back syndrome with radiating pain	12.1%	2.1%	5.1	(4.8-5.4)
65-79	T82	Obesity	27.3%	1.6%	17.6	(14.9-20.8)	T82	Obesity	17.0%	0.9%	17.0	(13.8-20.8)
	P76	Depressive disorder	24.6%	2.2%	9.7	(8.4-11.2)	T93	Lipid disorder	51.4%	7.3%	10.3	(9.5-11.2)
	T93	Lipid disorder	58.2%	9.7%	9.3	(8.6-10.0)	T90	Diabetes non-insulin dependent	29.4%	2.6%	8.5	(7.5-9.6)
	K86	Hypertension, uncomplicated	65.8%	11.3%	9.0	(8.4-9.7)	K86	Hypertension uncomplicated	63.1%	11.6%	8.5	(7.9-9.0)
	L95	Osteoporosis	25.7%	3.9%	6.3	(5.7-7.1)	Y85	Benign prostatic hypertrophy	32.3%	5.0%	6.3	(5.8-7.0)
80+	L91	Osteoarthrosis, other	28.1%	1.5%	19.6	(14.7-26.1)	T93	Lipid disorder	39.3%	2.4%	20.7	(15.3-28.1)
	T93	Lipid disorder	49.3%	3.8%	18.9	(15.7-22.8)	K86	Hypertension uncomplicated	68.6%	9.5%	15.5	(13.1-18.3)
	K86	Hypertension, uncomplicated	78.7%	14.6%	15.0	(13.5-16.7)	T90	Diabetes non-insulin dependent	28.1%	2.2%	12.0	(8.7-16.5)
	F92	Cataract	26.1%	1.7%	14.7	(11.2-19.3)	F92	Cataract	24.0%	2.0%	11.9	(8.5-16.7)
	T90	Diabetes, non-insulin dependent	25.0%	2.1%	10.3	(8.0-13.1)	Y85	Benign prostatic hypertrophy	41.0%	5.9%	8.7	(7.1-10.6)

*Abbreviations*: *ICPC 2* International Classification of Primary Care, *MM* multimorbidity, *OR* odds ratio, *CI* confidence interval.

All ORs were calculated by multilevel logistic regression and adjusted by visits and deprivation quintiles. PHCT was considered random variable. All ORs were statistically significant with a p-value <0.001.

The adjusted analysis, without stratification, showed a significant and positive association between multimorbidity and the following factors: sex (female, OR = 1.28; 95% CI: 1.27-1.29, compared with males); age (ranging from OR = 1.85 [95% CI: 1.81-1.88] in 25–44 age group to OR = 37.33 [95% CI: 36.03-38.68] in 80+ group, compared to 19–24 age group); socioeconomic status, increasing until Q4 (OR = 1.06 [95% CI: 1.04-1.07] in Q2, ranging to OR 1.10 [95% CI: 1.08-1.12] in Q4 and OR 1.07 [95% CI: 1.05-1.09] in Q5, compared to Q1); and number of visits (increasing from 1–2 visits, OR = 3.05 [95% CI: 3.01-3.10] to 11+ visits, OR = 26.22 [95% CI: 25.79-26.66], compared to 0 visits).

In multivariate analysis, women were more likely than men to have multimorbidity in all quintiles of the deprivation index until 65 years of age, compared with the least deprived group. Women of all age groups and men aged 25 to 65 years showed a significant positive association between deprivation quintiles and multimorbidity. The odds of multimorbidity increased as number of visits increased in all strata (Table 6).

**Table 6:** Factors associated with multimorbidity in 2010, stratified by sex and age group (N = 1,356,761)

	**Women**														
	**Age group (years)**														
	**19-24**			**25-44**			**45-64**			**65-79**			**80+**		
	**OR**	**95% CI**	***p-value***	**OR**	**95% CI**	***p-value***	**OR**	**95% CI**	***p-value***	**OR**	**95% CI**	***p-value***	**OR**	**95% CI**	***p-value***
*Deprivation index*															
*(ref. Least deprived)*			0.024			<0.001			<0.001			0.006			<0.001
Q2	1.10	1.01-1.19		1.06	1.03-1.10		1.07	1.03-1.11		1.01	0.93-1.10		1.20	1.05-1.35	
Q3	1.12	1.03-1.22		1.13	1.09-1.17		1.19	1.14-1.23		1.13	1.03-1.24		1.13	0.99-1.29	
Q4	1.07	0.98-1.17		1.11	1.07-1.15		1.22	1.17-1.27		1.15	1.05-1.27		1.26	1.09-1.46	
Most deprived	1.01	0.92-1.11		1.12	1.08-1.16		1.22	1.16-1.27		1.12	1.01-1.25		1.42	1.21-1.66	
*Number of visits (ref. 0)*			<0.001			<0.001			<0.001			<0.001			<0.001
1-2	2.05	1.88-2.25		2.29	2.22-2.37		3.55	3.34-3.68		4.88	4.49-5.29		4.00	3.50-4.57	
3-5	3.54	3.25-3.85		3.86	3.75-3.98		7.66	7.41-7.92		13.93	12.87-15.07		11.31	9.91-12.92	
6-10	5.77	5.30-6.29		6.83	6.63-7.04		16.09	15.54-16.66		36.26	33.47-39.28		26.20	22.97-29.88	
≥11	10.42	9.52-11.42		14.14	13.68-14.62		41.82	40.16-43.56		135.23	123.26-148.37		61.90	54.68-70.7	
Deviance of the model^$^ (empty vs. fitted) ICC^&^		50846.0 46932.4 4%			332571.7 295933.5 3%			269077.9 212131.5 3%			61840.2 44074.4 4%			23072.6 17583.1 4%	
	**Men**														
	**Age group (years)**														
	**19-24**			**25-44**			**45-64**			**65-79**			**80+**		
	**OR**	**95% CI**	***p-value***	**OR**	**95% CI**	***p-value***	**OR**	**95% CI**	***p-value***	**OR**	**95% CI**	***p-value***	**OR**	**95% CI**	***p-value***
*Deprivation index*															
*(ref. Least deprived)*			0.014			0.006			<0.001			0.293			0.166
Q2	0.96	0.87-1.06		1.04	1.00-1.07		1.05	1.02-1.09		1.03	0.95-1.11		1.06	0.90-1.26	
Q3	1.10	1.00-1.21		1.05	1.01-1.10		1.07	1.03-1.12		1.07	0.98-1.16		1.19	0.99-1.43	
Q4	1.01	0.92-1.12		1.03	0.99-1.07		1.10	1.05-1.14		1.08	0.98-1.18		1.24	1.03-1.49	
Most deprived	0.95	0.86-1.05		1.00	0.96-1.04		1.02	0.98-1.07		1.11	1.00-1.22		1.09	0.90-1.33	
*Number of visits (ref. 0)*			<0.001			<0.001			<0.001			<0.001			<0.001
1-2	2.05	1.89-2.23		2.43	2.36-2.51		3.46	3.36-3.57		4.23	3.90-4.58		4.59	3.80-5.55	
3-5	3.27	3.01-3.54		4.37	4.24-4.50		7.39	7.17-7.63		11.42	10.57-12.32		12.50	10.41-15.00	
6-10	5.39	4.95-5.87		7.62	7.39-7.87		15.02	14.53-15.52		28.85	26.69-31.19		33.10	27.54-39.78	
≥11	8.87	8.04-9.80		15.99	15.42-16.57		36.18	34.75-37.66		87.17	79.86-95.15		73.21	61.58-87.04	
Deviance of the model^$^ (empty vs. fitted) ICC^&^		41661.7 38924.6 4%			293079.3 259335.9 3%			273195.5 215314.5 3%			63565.4 47285.5 3%			13222.1 10076.1 4%	

Multilevel logistic regression*.

*Abbreviations*: *ref* reference, *Qi* deprivation quintiles (Q1, Least deprived to Q5. most deprived); *OR* odds ratio, *CI* confidence interval, *ICC* Intraclass Correlation Coefficient).

*Primary health care team (PHCT) was considered random variable. All ORs are adjusted for each factor presented in the table. OR represents the odds of multimorbidity for people who are in the corresponding category of the factor compared with those in the reference category.

^$^The deviance expresses the goodness of fit of the model, and was calculated using the -2*log likelihood. Empty vs. fitted model refers to the model without predictors vs. the model with the individual predictors.

^&^ICC measures the proportion of the total variance in the outcome that is attributable to the PHCT level after taking into account the individual predictors.

## Discussion

Our analysis in a large primary health care data sample shows that almost half of the urban population had multimorbidity, with the most frequent pattern of chronic conditions being the combination of uncomplicated hypertension and lipid disorder in adults older than 45 years. The prevalence was higher in women but increased with age in both sexes.

In patients younger than 80 years, multimorbidity prevalence was greater among women than men in all quintiles of the deprivation index. Moreover, multimorbidity increased as women’s socioeconomic status declined in all age groups. Overall, multimorbidity represented a high burden for primary health care, with a median of 8 times more patient visits than the healthier group. There was a highly positive association between higher demand for services and a diagnosis of multimorbidity.

A major strength of this study is the analysis of a large, high-quality database of primary care records, representative of a large population. In the context of a national health system with universal coverage, EHR data have been shown to yield more reliable and representative conclusions than those derived from survey-based studies [7,21]. Another important strength is the inclusion of all chronic diagnoses recorded in EHR; not restricting the analysis to a specific number of chronic conditions contributed to a more accurate analysis of the combinations of chronic conditions present in the population with multimorbidity. Finally, the stratification of the analysis by age and sex and the consideration of socioeconomic status as a potentially associated factor are strengths of this study.

The study has some limitations that could have influenced our results. First, chronic conditions could be underreported among patients who make fewer visits to primary care centres. For example, men younger than 65 years were less likely than other patient strata to visit their doctors. Beyond the retirement age in Spain (65 years), use of health services was similar for both men and women. Coinciding with the results of other studies, women reported making more visits to primary care centres than men, which can be attributed to their more proactive attitude toward prevention, to a worse state of health, or to the fact that they accompany others to doctors’ visits [22]. However, the stratified analysis allows accurate estimation within each age-sex stratum. Another aspect to be considered is that 29% of the Catalan population, especially people with higher socioeconomic status, buys private health insurance to supplement the universal health care services. However, research suggests this might have a greater impact on specialized care than on primary care services [23]. Furthermore, universal access to health care and medications makes it more likely that patients will seek care and receive a diagnosis [24,25]. On the other hand, there could be an over-representation of chronic diagnoses (e.g., hypertension, diabetes, hyperlipidaemia, etc.) that are included in the goals/incentives contracts of Catalan PHCT and therefore likely to be carefully recorded. Second, there is no universally accepted criterion for consensus classification of chronic conditions. This lack of accurate case definitions impedes the establishment of the true incidence/prevalence of a condition [26]. We observed that O’Halloran criteria have several misclassifications with respect to congenital diseases, benign tumours, and some cardiovascular disease risk factors (e.g., smoking) that are considered acute conditions [17]. In this sense, other authors have attempted to define a new classification system for chronicity according to the use of health resources [27] or the duration of acute and chronic care episodes [28]. Continued work is needed to establish a definition and measurement of chronicity that takes into account not only clinical criteria but also episode duration and the use of resources. Third, the study design did not allow the analysis of causal relationships in the associations observed between chronic conditions, and biological and psychosocial causal pathways have not been established [29]. Fourth, the information source for socioeconomic indicators was the Spanish Census, conducted every 10 years by the National Institute of Statistics. At the time the multimorbidity data was collected and initially analysed in 2010, the deprivation index was based on 2001 census data, the most recent data available at the time. Fifth, it would have been interesting to compare our urban data on multimorbidity with rural areas of Catalonia; however, the MEDEA index is useful only as an instrument to detect areas of cities with unfavourable socioeconomic characteristics. More research is required to develop a deprivation index that can be applied in rural areas. Finally, a residual confounding cannot be completely excluded, because of epidemiological factors or other health determinants that were not considered in this study, such as environmental factors.

The estimated multimorbidity prevalence in our sample is higher than in other European studies [3,5,10,11,30], perhaps because of the analysis of a greater number of chronic conditions in our study than in most other published studies [31]. Nonetheless, the patterns of multimorbidity were similar to those observed by others [32]. The small differences were likely due to the type of statistical analysis (dyad or triad combinations, factor analysis or cluster analysis), age ranges of the study population, and the measures of multimorbidity and morbidity burden [33,34].

As in other studies, multimorbidity was more prevalent among women [3,5,10,11]. This could be due to longer life expectancy and worse health status compared to men, differences that are due to both biological and social factors [35]. In addition, sex is a social determinant that influences health status, health behaviours and the use of health services [36-38].

Some evidence has indicated that lower socioeconomic status is associated with a higher probability of multimorbidity [2,10,11]. Our study observed a positive association only in women and in men aged 25 to 65 years, but this was not a trend. Universal coverage could attenuate socioeconomic inequalities in health care, such as use of primary health care services.

Contrary to what we might anticipate, multimorbidity patterns were similar between age groups in both men and women over 45 years of age. The study of multimorbidity stratified by sex and age groups revealed some clinical phenomena that merit review. For example, we note that a circulatory-endocrine-metabolic pattern (dyslipidaemia, obesity and hypertension) appears earlier in men (beginning at 25 years of age) than in women (beginning 20 years later), and that psychological disorders (especially anxiety and depressive disorders) are the most prevalent chronic conditions among younger women. Both findings have implications for public health. Given the need for earlier detection of circulatory-endocrine-metabolic problems in men and the reported association between anxiety disorders and physical disorders [39], recognizing a patient’s potential for future physical disease can help to design intensive strategies for primary care prevention and treatment. On the other hand, the present study did not find a preponderance of respiratory illness, despite studies emphasizing the health impact of air pollution in urban environments [40].

If awareness of multimorbidity patterns by strata allows the design of individualized strategies to prevent chronicity, the clinician would have an additional tool for predicting the need to encourage personalized disease prevention and health promotion activities. Similarly, this awareness could lead to the development of Clinical Practice Guidelines to ensure adequate quality and safety indicators in multimorbidity prevention, diagnosis and treatment [41].

Women in urban areas, and specifically the most socioeconomically deprived, are more likely to have multimorbidity than men. These inequalities related to sex and deprivation quintiles are important to health policy, particularly considering the decline in public health resources in some European countries because of the economic crisis of the past several years [42]. Health policies usually focus on addressing inequalities based on race/ethnicity and socioeconomic status in urban settings, but must also consider factors such as geographical characteristics and gender [43]. To avoid inequalities in health status, limited health resources must be adjusted according to multimorbidity burden and socioeconomic status. In this sense, health economics experts will be forced to return to the longstanding argument about whether national health systems should increase and enhance primary health care services, typically at the expense of hospital care [44].

## Conclusions

With the inclusion in the analysis of all chronic conditions, multimorbidity affects almost half of the adult population in urban areas of Catalonia. There are differences in multimorbidity prevalence based on a patient’s sex, age group and socioeconomic status. Multimorbidity patterns differ by life-stage and sex; however, circulatory-endocrine-metabolic patterns remain constant within the most prevalent pairs and triads of chronic conditions after 45 years of age. Women younger than 80 years had greater prevalence of multimorbidity than men. In addition, multimorbidity prevalence increased as women’s socioeconomic status declined in all age groups. Patients with multimorbidity increased the burden on primary care services. Identification of multimorbidity patterns by life-stage supports better clinical management and prioritization by public health systems.

## Abbreviations

CHI: Catalan Health Institute; PHCT: Primary health care teams; SIDIAP: System for the Development of Research in Primary Care; EHR: Electronic health records; ICD 10: International classification of diseases; ICPC: International Classification of Primary Care; IDIAP: Institut Universitari d’Investigació en Atenció Primària.

## Competing interests

The authors declare that they have no competing interests.

## Authors’ contributions

All authors contributed to the design of the study, revised the article, and approved the final version. CV, QFB, JMV drafted the study protocol and obtained the funding. TR, AR, CV, QFB and JMV contributed to the analysis and interpretation of data. CV, QFB, JMV, TR and AR wrote the first draft, and all authors contributed ideas, interpreted the findings and reviewed rough drafts of the manuscript.

## Pre-publication history

The pre-publication history for this paper can be accessed here:

http://www.biomedcentral.com/1471-2458/14/530/prepub

## Supplementary Material

Additional file 1 List of the five most prevalent chronic conditions, stratified by sex and age in 2010 (N = 1,356,761). Click here for file
