# Supplementary material for: Burden of multimorbidity, socioeconomic status and use of health services across stages of life in urban areas: a cross-sectional study
Source: BMC Public Health. 2014 May 29;14:530. doi: 10.1186/1471-2458-14-530 (PMC4060853; doi:10.1186/1471-2458-14-530)
Supplement: Additional file 1 — List of the five most prevalent chronic conditions, stratified by sex and age in 2010 (N = 1,356,761). [file 1471-2458-14-530-S1.docx]

**Additional file 1. List of the five most prevalent chronic conditions in 2010, stratified by sex and age (N=1,356,761)**

Abbreviations: n, total number of patients in each stratum; ICPC 2, International Classification of Primary Care; CI, confidence interval.
